# Prevalence and correlates of sleep quality in the Chinese college students with migraine: a cross-sectional study

**DOI:** 10.3389/fnbeh.2022.1037103

**Published:** 2022-11-01

**Authors:** Jiayu Zhao, Yu Cen, Jiaming Yang, Chang Liu, Yajie Li, Zhen Ren, Yun Xiao, JinLong He, Jing Luo, Yunling Zhong, Wenxiu Luo, Jing Wu, Jiaming Luo

**Affiliations:** ^1^Department of Neurology, Affiliated Hospital of North Sichuan Medical College, Nanchong, China; ^2^School of Psychiatry, North Sichuan Medical College, Nanchong, China; ^3^Mental Health Center, Affiliated Hospital of North Sichuan Medical College, Nanchong, China; ^4^Mental Health Center, Southwest Petroleum University, Nanchong, China

**Keywords:** migraine, sleep quality, mobile phone addiction, anxiety, depression, college students

## Abstract

**Background:** Migraineurs are often plagued by sleep disorders. The university student population is high in number and is more vulnerable to migraines. However, no study has analyzed the sleep quality of students with migraine and related contributing factors.

**Objective:** We used the Pittsburgh Sleep Quality Index (PSQI) scale to assess the sleep of migraine patients among college students and to explore the influencing factors of sleep quality.

**Methods:** We performed primary screening for migraine using the ID-migraine screening, and further assessed headache characteristics, sleep, anxiety, depression, and mobile phone addiction in college students with positive primary screening, then diagnosed migraine according to the third edition of* the International Classification of Headache Disorders* (ICHD-3). Finally, we analyzed the factors influencing sleep quality using Binary Logistic Regression Analysis. Those with scores greater than 5 points on the PSQI scale were believed to have poor sleep quality.

**Results:** The prevalence of migraine was 6.6%. A total of 545 migraineurs were eventually included in the analysis, the incidence of poor sleep quality was 64.04%. The three factors of experiencing aura (OR = 2.966, 95%CI = 1.756–5.010, *P* < 0.05), anxiety (OR = 2.778, 95%CI = 1.434–5.382, *P* < 0.05), and high Mobile phone addiction index (MPAI) score (OR = 1.025, 95%CI = 1.002–1.049, *P* < 0.05) contributed enormously to poor sleep quality. Moreover, the factors of aura symptoms (OR = 3.796, 95%CI = 2.041–7.058, *P* < 0.05), anxiety (OR = 3.146, 95%CI = 1.473–6.719, *P* < 0.05), and MPAI score (OR = 1.028, 95%CI = 1.002–1.054, *P* < 0.05) influenced the sleep quality of female migraineurs rather than male migraineurs.

**Conclusions:** The incidence of poor sleep quality is high among university students with migraine. Aura symptoms, anxiety, and high MPAI score influence the sleep quality of migraineurs, especially females. The proposal of prevention and intervention measures is of great importance to the physical and mental health of students with migraine.

**Clinical Trial Registration:** identifier ChiCTR1800014343.

## Introduction

Migraine is one of the most common types of primary headaches with high morbidity and disability, and it is reported that the current prevalence of migraine worldwide is 10% to 18% (Yu et al., 2012; Al-Hashel et al., 2014; Woldeamanuel and Cowan, 2017). Migraine and sleep disorders are common serious chronic conditions, with a high prevalence among the general population (Lipton et al., 2003; Stang et al., 2004). The research results of Viticchi et al. (2020) showed that headache sufferers with high PSQI scores experienced more frequent headache attacks and required more medications to ease the pain, so their life quality was greatly affected. An African study of migraine found that 60.5% of migraine sufferers had poor sleep quality (Morgan et al., 2015). Another research on students with migraine indicated that 85.9% of them had poor sleep quality (Walters et al., 2014). Migraine sufferers are more likely to have poor sleep quality, which seriously affects their life quality and learning ability, as well as adds to the economic burden.

Previous studies showed that compared with non-migraine people, migraine sufferers had worse sleep quality (Engstrøm et al., 2014; Duman et al., 2015), which was associated with an increased frequency of migraine attacks and chronicity (Sadeghniiat et al., 2013; Lin et al., 2016; Lucchesi et al., 2016; Song et al., 2018). The relationship between migraine and sleep is quite complicated. On the one hand, examples of insomnia, poor sleep maintenance, and sleeping for too long or too short can induce migraine and increase the frequency and intensity of headache attacks (Fernández-de-Las-Peñas et al., 2018; Song et al., 2018), resulting in more associated negative repercussions (Cho et al., 2020). On the other hand, frequent headache attacks or acute headaches can lead to sleep disorders (Lin et al., 2016; Fernández-de-Las-Peñas et al., 2018). An Iranian survey of headaches in children (aged 6–12) showed that children with migraine were more liable to sleep disorders with the attack of severe headaches, and the average intensity of headache was significantly related to sleep quality (*P* < 0.05; Cheraghi et al., 2018). It has been discovered that a possible link may exist between migraine and the pathogenesis of sleep disorders, and some central nervous system structures and neurotransmitters may be acting (Dodick et al., 2003; Holland et al., 2018; Vgontzas and Pavlović, 2018). A Mata analysis report displayed that psychological sleep intervention imposed on migraine sufferers in multiple studies helped improve several sleep results (such as sleep duration and efficiency, hypersomnia) and headache frequency, despite the contradictory result of influences on headache intensity (Sullivan et al., 2019). Chronic migraine sufferers with poor sleep quality are more prone to pain and depression (Garrigós-Pedrón et al., 2019).

College students are a group with a large population size andthey are more vulnerable to migraines, and as a result, some students may suffer from headaches. Al-Hashel et al. (2014) took advantage of the ID-migraine screening questionnaire to survey the prevalence of migraine at Kuwait University and discovered that 27.9% of the subjects were diagnosed with migraine. A study in China investigating the prevalence of migraine among university students showed that the overall prevalence was 7.91% and females (9.84%) were more prone to migraine than males (4.64%; Gu and Xie, 2018). Another survey of migraine among university students in Harbin showed that the prevalence of migraine was 9.0% and the percentage increased with age (Wang et al., 2015b). Previous studies have demonstrated the high prevalence of migraine among Chinese university students, which substantially affects their life and study. Nevertheless, these students have little knowledge of migraine.

Research in China has only focused on the prevalence of migraine among university students, but previous studies showed that migraine, together with poor sleep quality, had severe impacts on the sufferers’ life quality and learning ability. Little research was done on the prevalence of migraine combined with poor sleep quality among Chinese students, and the clinical features of sufferers with varied sleep quality and the factors affecting sleep quality were still unclear. Therefore, this study aims to investigate the sleep quality of Chinese university students with migraine and discuss the latent factors influencing sleep quality, which will make for the proposal of corresponding measures to mitigate headache attacks and improve sleep quality, to enhance students’ learning ability and life quality.

## Methods

### Participants and procedure

This survey was conducted at a university in Nanchong city from July 2018 to July 2019. It was a cross-sectional study on migraine, mainly investigating the prevalence and clinical features of migraine, sleep and life quality, and learning ability of students. Research subjects were students from North Sichuan Medical College and with the cluster sampling method, data were obtained by filling in the questionnaire face to face.

The study was divided into three stages. In the first stage, the questionnaires were issued to the research participants and they were asked whether recurrent headaches happened to them (twice or more than twice a year). Future research applied to the participants who answered “yes,” excluding those who answered “no.”

In the second stage, ID-migraine screening was conducted with three simple questions: (1) are there any social, occupational, learning or daily activities affected by headaches in the past 3 months?; (2) do you have a stomach upset, nausea or vomiting during a headache attack?; and (3) are you afraid of light with a headache? ID-migraine is a simple but effective headache screening tool and is often used to diagnose migraine (Domingues et al., 2011; Al-Hashel et al., 2014; Wang et al., 2015b). ID-migraine showed good sensitivity (0.84, 95%CI 0.75–0.90) and specificity (0.76, 95%CI 0.69–0.83; Cousins et al., 2011). The Chinese version of ID-migraine had a sensitivity of 0.81 (95%CI 0.80–0.82), a specificity of 0.68 (95%CI 0.66–0.69), as well as a diagnostic odds ratio (DOR) of 17.03 (95%CI 9.94–29.18; Wang et al., 2015a). If two or more than two answers to the three questions were “yes,” then the initial screening of the participant was considered positive, otherwise negative and no further research would be carried out.

The participants with positive initial screening results were asked by phone or e-mail whether they were willing to take part in the third stage of the survey. In the third stage, the participants were required to fill in a self-made questionnaire which included general demographic data and clinical features, Visual Analogue Scale (VAS), Headache Impact Test-6 (HIT-6) scale, Hamilton Anxiety and Depression Scales (HAMA and HAMD), Pittsburgh Sleep Quality Index (PSQI) scale, Mobile Phone Addiction Index (MPAI) scale, and others. The research members were made up of professional neurologists in the Affiliated Hospital of North Sichuan Medical College, graduate students and undergraduate students majoring in clinical medicine at North Sichuan Medical College. Before the survey, each member was trained in professional knowledge and questionnaire-related questions and they all passed relevant tests. A unified standard to collect data was also made, insomuch that each member was able to collect data and the collected data were qualified. During data collection, all researchers strictly adhered to the Standard Operation Procedure (SOP) and before the research, they fully understood latent problems and made relevant preparations.

### Diagnostic code (DC) and exclusion criteria

In this study, a diagnosis of migraine was made according to the third edition of* the International Classification of Headache Disorders* (ICHD-3; International Classification of Headache, 2018). The inclusion criteria were: (1) two professional neurologists confirmed that the migraine sufferers met the diagnostic code and classification criteria of the ICHD-3; (2) no serious physical diseases; (3) no acute or chronic infection in the past 1 month; and (4) not taking migraine medication or antidepressant (antianxiety) drugs for at least 2 weeks. The exclusion criteria were: (1) sufferers with other systemic diseases or brain diseases; (2) sufferers with secondary headaches or other chronic diseases; (3) sufferers who cannot understand the contents of the scales because of related dysfunction; (4) sufferers who already had sleep disorders or mental illness before headache; (5) sufferers who are taking migraine medication, or antianxiety or antidepressant drugs; and (6) sufferers who refuse to sign the informed consent form.

### Data collection and measurement

After understanding the goals of the questionnaire, the participants needed to complete the questionnaire. General demographic data and clinical features include sex, age, nationality, height, weight, qualification, major, grade, living allowance, marital status, smoking and drinking, past illness, long-term medication history, headache treatment, family history, headache situation (starting age, pathogenesis, cause, incentive, headache pattern/location/nature/duration, degree, aggravating and mitigating factors, aura symptoms, accompanying symptoms, headache mitigation status, headache attack laws), number of days with headache per month, painkiller use, among others.

The degree of headache was assessed using the VAS, with 0 points for no pain, 1–3 points for mild pain, 4–6 points for moderate pain, 7–9 points for severe pain and 10 points for being the most painful (Aun et al., 1986).

Headache-related effects were measured with the HIT-6 scale. The scale covers headache intensity, social function, cognitive function, psychological abnormalities and vitality, which can better assess the impact of headaches on daily life. Clinically, the scale is widely used to screen and monitor the treatment of headache patients, including migraine sufferers (De Diego and Lanteri-Minet, 2005; Nachit-Ouinekh et al., 2005; El Hasnaoui et al., 2006; Yang et al., 2011). Scores less than 49 indicate small or no impact, 50–55 points indicate limited impact, 56–59 points indicate major impact and scores over 60 indicate significant impact.

PSQI scale is suitable for assessing the sleep quality of the general population, as well as the people with sleep disorders and mental disorders, so it is widely used to assess the sleep quality of migraine sufferers. The scale has a sensitivity of 89.6% and a specificity of 86.5% (kappa = 0.75, *P* < 0.001; Buysse et al., 1989). With PSQI (Chinese version), this survey assessed the sleep quality of migraine sufferers within 1 month. Whoever obtains more than 5 points from the scale is believed to have poor sleep quality, and less than or exactly 5 points is believed to have good sleep quality (Song et al., 2018; Wu et al., 2020). Generally, the higher the points are, the worse the sleep quality is.

HAMA (Hamilton, 1959) and HAMD (Hamilton, 1959) are widely used to assess anxiety, depression and its severity. The 24-item version of HAMD was utilized in this study. The participants were evaluated by rigorously trained professionals. Whoever obtains 14 or more than 14 points on the HAMA scale is considered anxious, and those who obtain more than 20 points on the HAMD scale are regarded as depressed.

MPAI scale, developed by Louis (2008), is used to characterize mobile phone addiction symptoms among adolescents and students. The passage referred to in this study is the Chinese version translated by Huang et al. (2014), which has fair reliability and validity (Huang et al., 2014; Zhang et al., 2021). The four mobile phone addiction symptoms are inability to control craving, feeling anxious and lost, withdrawal/escape and productivity loss. The higher the scores on the scale are, the heavier the dependence on the mobile phone is. Addiction criteria are: with Questions 3, 4, 5, 6, 8, 9, 14, and 15 as screening questions, whoever answers with more than five “yes” is considered addicted to mobile phone or otherwise (Wang et al., 2014).

### Statistical analysis

Data analysis was conducted with SPSS26.0 and Forest plotting was conducted with GraphPad Prism8.0.2. Kolmogorov-Smirnov test (K-S test) was used to test the normality of distribution features of measurement data. Measurement data in accordance with normal distribution were expressed as mean ± standard deviation ($\bar{\chi }$ ± s) and student tests of two independent samples were implemented to realize group comparisons. Measurement data falling short of normal distribution were demonstrated as median [interquartile range; M (IQR)] and group comparisons were realized with the nonparametric test. Categorical data were displayed in percentage terms (%) and the χ^2^ test was used to carry out group comparisons. To determine the predictive factors of poor sleep quality, univariate and binary logistic regression analyses were utilized, respectively. The predictive factors of poor sleep quality were illustrated with 95% confidence interval (95%CI) and odds ratio (OR) and all the *p*-values were two-tailed with a significance level of 0.05.

## Results

### Survey result

A total of 9,057 ID-migraine initial screening questionnaires were distributed and 8,783 valid questionnaires were recovered, with an effective response rate of 96.97%. A total of 1,037 initial screening results were positive, and 1,007 students participated in the third stage of information collection, with a collection rate of 97.11%. Eventually, 579 participants were diagnosed with migraine and the prevalence was 6.6% (579/8,783), which had been reported in the previous studies (Luo et al., 2021). To further explore the sleep quality of migraine sufferers, 34 questionnaires with incomplete answers to the PSQI scale were excluded, and thereby 545 students with migraine were included in the study. Relevant analysis showed that the incidence of poor sleep quality among migraine sufferers was 64.04%, among whom 284 were schoolgirls (81.38%).

### Demographic characteristics, headache-related features, accompanying psychiatric problems, and MPAI situations of Migraineurs

Of the 545 migraine sufferers included in this study, their average age was 19.59 ± 1.59 years old and 437 of them were schoolgirls (80.18%). The migraine sufferers who obtained more than 5 points from the PSQI scale were classified into the group of “poor sleep quality” and those who obtained 5 or less than 5 points were classified into the group of “good sleep quality.” Analysis of general demographic data, headache features, accompanying psychiatric problems and MPAI situations of migraine sufferers in the two groups were implemented and the detailed results are shown in Table 1. Between the two groups, there were significant discrepancies in the aura, headache frequency, HIT-6 score, anxiety, depression, MPAI total score and its sub-items of inability to control craving, feeling anxious and lost, withdrawal/escape and productivity loss (*P* < 0.05). Sufferers’ headache frequency, HIT-6 score and MPAI score in the poor sleep quality group were higher than those in the good sleep quality group. Likewise, the students in the poor sleep quality group also had higher ratios of anxiety, depression, and aura symptoms. However, in terms of age, sex, Body mass index (BMI), family history, duration of headache, headache intensity, and mobile phone addiction, there are no statistical differences between the two groups (*P* > 0.05).

**Table 1:** Sociodemographic characteristics of surveyed participants.

**Characteristics**	**Migraine with poor sleep quality (*n* = 349)**	**Migraine without poor sleep quality (*n* = 196)**	***P* value**
**Demographic characteristics**
Age	19.63 ± 1.53	19.53 ± 1.69	0.467
BMI	20.34 ± 2.78	20.39 ± 3.13	0.839
Sex			0.352
Female	284 (81.4%)	153 (78.1%)	
Male	65 (18.6%)	43 (28.9%)	
Smoking			0.355
Yes	12 (3.5%)	4 (2.1%)	
No	334 (96.5%)	190 (97.9%)	
Drinking			0.316
Yes	17 (5.0%)	6 (3.1%)		
No	326 (95.0%)	186 (96.9%)	
Family history			0.896
Yes	125 (56.8%)	78 (56.1%)	
No	95 (43.2%)	61 (43.9%)	
**Headache-related features**			
Aura symptoms			0.000
Yes	268 (80.5%)	115 (61.5%)	
No	65 (19.5%)	72 (38.5%)	
Headache frequency	2.00 (1.00, 5.00)	1.00 (1.00, 3.00)	0.000
Duration of headache			0.168
<1 h	183 (54.1%)	90 (47.9%)	
>1 h	155 (45.9%)	98 (52.1%)	
VAS score for headache intensity			0.590
Mild	154 (44.5%)	81 (41.8%)	
Moderate	175 (50.6%)	104 (53.6%)	
Severe	17 (4.9%)	9 (4.6%)	
HIT-6 score	55.18 ± 8.62	52.90 ± 8.69	0.005
**Accompanying psychiatric problems**		
Anxiety (HAMA score ≥14)	111 (33.33%)	19 (10.05%)	0.000
Depression (HAMD score > 20)	72 (20.75%)	10 (5.21%)	0.000
**MPAI**			
Mobile phone addiction			0.162
Yes	50 (15.4%)	20 (10.9%)	
No	275 (84.6%)	163 (89.1%)	
MPAI total score	44.76 ± 10.80	40.80 ± 10.90	0.000
Uncontrolled	18.82 ± 5.02	16.98 ± 4.92	0.000
Avoidant	8.43 ± 2.75	7.93 ± 3.02	0.058
Withdrawal	9.54 ± 3.45	8.40 ± 3.41	0.000
Inefficiency	8.27 ± 2.65	7.25 ± 2.43	0.000

MPAI scale survey results: 45 students (17.0%) in the poor sleep quality group spent an average of over 8 h on their mobile phone per day, which is much more than the 17 students (11.5%) in the good sleep quality group. Students with a mobile phone for more than 5 years in the good sleep quality group accounted for 35.8%, higher than those in the poor sleep quality group (32.8%). Students who spent an average of 51–100 yuan on mobile phones per month took up the largest proportion (44.1%) in the poor sleep quality group, whereas their counterparts mostly spent less than 50 yuan (48.7%). As for the main motives for mobile phone use, students in both groups were for interpersonal needs and recreation, with the respective proportions of 23.1% and 49.0% in the poor sleep quality group and those of 26.6% and 48.2% in the other group. However, the aforementioned items made no statistical difference (*P* > 0.05).

### Comparison of PSQI scale scores between Migraineurs with and without aura symptoms

Based on the DC of migraine sufferers with and without aura (MA and MOA) from ICHD-3, students with migraine in this study were classified into two groups, one group being students with aura symptoms and the other group without aura symptoms. The total and sub-item PSQI scores are as follows: sleep quality ($\bar{\chi }$ ± s; 1.19 ± 0.762 vs. 1.19 ± 0.711, *P* = 0.977), sleep latency (1.22 ± 0.950 vs. 1.19 ± 1.036, *P* = 0.759), sleep duration (1.13 ± 0.775 vs. 1.27 ± 0.777, *P* = 0.144), sleep disturbance (1.03 ± 0.547 vs. 1.17 ± 0.529, *P* = 0.037), use of sleeping medication (0.08 ± 0.390 vs. 0.03 ± 0.162, *P* = 0.056), daytime dysfunction (1.88 ± 0.851 vs. 1.67 ± 0.827, *P* = 0.043), sleep efficiency (median; 0 vs. 0, *P* = 0.957), PSQI total score (6.75 ± 3.107 vs. 6.68 ± 2.537, *P* = 0.851). Migraine sufferers with aura symptoms had a higher average of sleep disturbance than those without aura symptoms who, on the other hand, had a higher score of daytime dysfunctions than migraine sufferers with aura symptoms, both of which were statistically significant (*P* < 0.05). Though the average sleep duration of migraine sufferers without aura symptoms (6.75 ± 0.937 h) was longer than those with aura symptoms (6.62 ± 1.066 h), the difference was not statistically significant (*P* > 0.05), see Table 2.

**Table 2:** Total and sub-item PSQI scores among participants with Migraine with/without aura.

	**Migraine without aura (*n* = 470, 86.2%)**	**Migraine with aura (*n* = 75, 13.8%)**	***P* value**
Subjective sleep quality	1.19 ± 0.762	1.19 ± 0.711	0.977
Sleep latency	1.22 ± 0.950	1.19 ± 1.036	0.759
Sleep duration	1.13 ± 0.775	1.27 ± 0.777	0.144
Habitual sleep efficiency	0.00 (0.00, 0.00)	0.00 (0.00, 0.00)	0.957
Sleep disturbance	1.03 ± 0.547	1.17 ± 0.529	0.037
Use of sleeping medication	0.08 ± 0.390	0.03 ± 0.162	0.056
Daytime dysfunction	1.88 ± 0.851	1.67 ± 0.827	0.043
Total	6.75 ± 3.107	6.68 ± 2.537	0.851

### Analysis of factors that influence the sleep quality of migraine sufferers

Univariate logistic regression analysis was performed on the items with statistical differences between the two groups in Table 1 and the items were: aura symptoms, headache frequency, HIT-6 score, anxiety, depression, MPAI score, and sex. The detailed analysis is shown in Table 3. It is discovered that sex had nothing to do with the sleep quality of migraine sufferers (*P* > 0.05) and the sufferers with aura symptoms (OR = 2.581, 95%CI = 1.730–3.852, *P* < 0.01), anxiety (OR = 4.227, 95%CI = 2.491–7.174, *P* < 0.01), and depression (OR = 4.765, 95%CI = 2.396–9.476, *P* < 0.01) were more liable to poor sleep quality. Moreover, migraine sufferers with high HIT-6 scores (OR = 1.031, 95%CI = 1.009–1.054, *P* < 0.01), headache frequency (OR = 1.099, 95%CI = 1.035–1.167, *P* < 0.01), and MPAI scores (OR = 1.036, 95%CI = 1.017–1.055, *P* < 0.01) were more likely to have poor sleep quality.

**Table 3:** Univariate logistic regression analysis of contributing factors related to the sleep quality in participants with migraine.

**Factors**	**ß**	**OR (95%CI)**	***P* value**
Sex (male)	−0.205	0.814 (0.528, 1.255)	0.352
Aura symptom	0.948	2.581 (1.730, 3.852)	0.000
HIT-6 score	0.031	1.031 (1.009, 1.054)	0.005
Headache frequency	0.095	1.099 (1.035, 1.167)	0.002
Depression	1.561	4.765 (2.396, 9.476)	0.000
Anxiety	1.442	4.227 (2.491, 7.174)	0.000
MPAI score	0.035	1.036 (1.017, 1.055)	0.000

Such factors as sex, aura symptom, HIT-6 score, headache frequency, depression, anxiety, and MPAI score were included in binary logistic regression analysis as independent variables, in Table 4. The results showed that aura symptom (ß = 1.087, OR = 2.966), anxiety (ß = 1.022, OR = 2.778), and MPAI score (ß = 0.025, OR = 1.025) contributed significantly to the poor sleep quality of students with migraine (*P* < 0.05), but sex (ß = −0.140, OR = 0.869), HIT-6 score (ß = 0.001, OR = 1.001), headache frequency (ß = 0.044, OR = 1.045), and depression (ß = 0.271, OR = 1.312) had no impact on sleep quality.

**Table 4:** Multivariate logistic regression analysis of factors associated with sleep quality in migraine patients.

**Factors**	**ß**	**OR (95%CI)**	***P* value**
Sex (male)	−0.140	0.869 (0.483, 1.563)	0.639
Aura symptom	1.087	2.966 (1.756, 5.010)	0.000
HIT-6 score	0.001	1.001 (0.973, 1.029)	0.963
Headache frequency	0.044	1.045 (0.983, 1.112)	0.157
Depression	0.271	1.312 (0.568, 3.028)	0.525
Anxiety	1.022	2.778 (1.434, 5.382)	0.002
MPAI score	0.025	1.025 (1.002, 1.049)	0.036
Constant	−1.721	0.179 (-)	0.054

**Figure 1:**
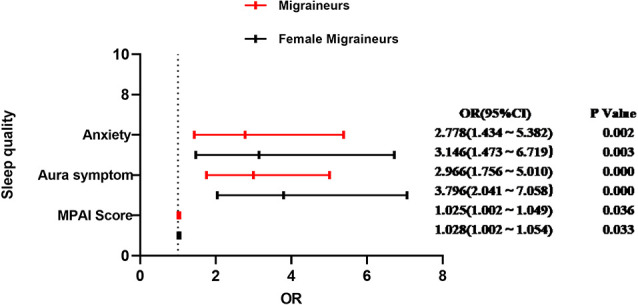
Influencing factors of sleep quality in migraine and female migraine patients.

A further binary logistic regression analysis of the factors that influence the sleep quality of male and female migraine sufferers was conducted and the results are displayed in Tables s 5 and 6. The factors of aura symptom (ß = 0.511, OR = 1.667; ß = 1.334, OR = 3.796), anxiety (ß = 0.671, OR = 1.957; ß = 1.146, OR = 3.146), and MPAI score (ß = 0.015, OR = 1.015; ß = 0.027, OR = 1.028) showed no influence on the sleep quality of male participants (*P* > 0.05), but they affected the sleep quality of female participants and the effect was of statistical significance (*p* < 0.05). Factors influencing sleep quality in migraine and female migraine patients are shown in Figure 1.

**Table 5:** Multivariate logistic regression analysis of factors associated with sleep quality in male migraine patients.

**Factors**	**ß**	**OR (95%CI)**	***P* value**
Aura symptom	0.511	1.667 (0.587, 4.728)	0.337
HIT-6 score	0.005	1.005 (0.951, 1.062)	0.869
Headache frequency	0.019	1.019 (0.919, 1.130)	0.719
Depression	0.053	1.055 (0.152, 7.298)	0.957
Anxiety	0.671	1.957 (0.462, 8.289)	0.362
MPAI score	0.015	1.015 (0.952, 1.083)	0.642
constant	−1.149	0.317 (-)	0.516

**Table 6:** Multivariate logistic regression analysis of factors associated with sleep quality in female migraine patients.

**Factors**	**ß**	**OR (95%CI)**	***P* value**
Aura symptom	1.334	3.796 (2.041, 7.058)	0.000
HIT-6 score	−0.001	0.999 (0.966, 1.032)	0.939
Headache frequency	0.063	1.065 (0.986, 1.151)	0.109
Depression	0.305	1.357 (0.530, 3.472)	0.525
Anxiety	1.146	3.146 (1.473, 6.719)	0.003
MPAI score	0.027	1.028 (1.002, 1.054)	0.033
constant	−1.992	0.136 (-)	0.053

## Discussion

This study reveals that the prevalence of migraine among students is 6.6%, which is lower than that shown by previous studies in China on the general population and students (Yu et al., 2012; Wang et al., 2015b; Gu and Xie, 2018). The reasons may be attributed to the environmental temperature (Yang et al., 2015; Li et al., 2019), as well as the larger sample size of this study. Compared with the prevalence of migraine abroad, the prevalence obtained from this study is far lower than in Europe and America (Johnson et al., 2014) and African regions (Morgan et al., 2015), but similar to that in South Korea (Song et al., 2018), which may be ascribed to the different research methods and higher response rate in this study (96.97%).

The study shows that the incidence of poor sleep quality among students with migraine at North Sichuan Medical College is 64.04%. The incidence of poor sleep quality among foreign students with migraine is 85.9% (Walters et al., 2014), and the incidence of poor sleep quality among students in Jilin Province, China is 31.0% (Li et al., 2020). Among the general students in China, those with migraine are more liable for poor sleep quality. On the one hand, students with migraine are often negatively affected by headache attacks and aura symptoms, resulting in worse sleep quality, and meanwhile they are often plagued by such psychiatric complications as anxiety and depression (Peres et al., 2017), which are likely to contribute to sleep disorders (Kim et al., 2017). On the other hand, subjects in the study are medical students and faced with hectic work schedules and academic stress, they have to stay up late and lack physical exercise, thus inviting worse sleep quality. By analyzing the previous studies, it is found that the incidence of poor sleep quality of foreign students with migraine is higher than that in China, which may be caused by differences in research methods and population groups. Whether the incidence may be influenced by living habits and personality is worth further exploration. Moreover, compared with the prevalence of poor sleep quality in outpatients with migraine in Southwest China (61.61%; Zhu et al., 2013), the incidence among students is still relatively high, which may be ascribed to greater academic pressure and different sleeping habits. The incidence of poor sleep quality of migraine sufferers in the research of Cho et al. (2020) is higher than that in this study; the incidences in the studies of Duman et al. (2015) and Morgan et al. (2015) are roughly equivalent to that in this study; the incidence in the research of Song et al. (2018) is lower than that in this study. The different incidences are possibly due to the differences in research methods, subjects, regions, among others.

Migraine sufferers in good and poor sleep quality groups manifest differences in aura symptoms, headache frequency, and HIT-6 score. Migraine sufferers in the poor sleep quality group tend to have a higher proportion of aura symptoms and headache frequency, which means that aura symptoms and headache frequency affect sleep quality to a certain extent, exactly in line with the research results of Walters et al. (2014) and Song et al. (2018). Migraine sufferers in the poor sleep quality group obtain higher HIT-6 scores, indicating that the migraine sufferers with poor sleep quality are more easily affected by headaches in daily life and consequently they have poor life quality, which accords with the opinions of Cho et al. (2020). The discovery that headache duration and headache intensity in the two groups have no significant difference is in accordance with the research results of Song et al. (2018), indicating that the duration of headache and headache intensity may have no impact on sleep quality, but the discovery contradicts the research results of Yalinay Dikmen et al. (2015). To the best of our knowledge, the evidence itself from available studies on the effect of headache intensity on sleep quality is conflicting, so further research is needed to discover the relationship between the two.

We compared the sleep quality of MA and MOA and discovered that MOA sufferers had higher total PSQI scores than MA sufferers, which, however, is not statistically significant. MA and MOA sufferers share the same incidence of poor sleep quality. The discrepancies in sleep disturbance and daytime dysfunction are of statistical significance (*P* < 0.05). MA sufferers obtain higher scores in sleep disturbance, whereas MOA sufferers get higher scores in daytime dysfunction. According to Vgontzas et al. (2020), physiological changes in the aura stage may cause sleep changes, and MA sufferers usually undergo more psychological disorders (Luo et al., 2021), they are likely to obtain higher scores in sleep disturbance. However, this study did not take into account the specific duration of aura symptoms, aura symptoms happening before or concurrent with headache, and concrete psychological disorders of the sufferers, which may explain the same incidence of poor sleep quality for both MA and MOA sufferers. We speculate that when the aura symptoms last long or happen concurrently with headache, sleep quality is significantly influenced, but no literature buttresses this speculation. Therefore, we can further study the impact that the duration of aura symptoms exerts on sleep quality, insomuch that the relationship between the two can be discovered.

This study conclusively identified several risk factors for poor sleep quality of Chinese students with migraine. Migraine sufferers with aura symptoms were 2.966 times more likely to have poor sleep quality than those without aura symptoms. In fact, fMRI (functioning magnetic resonance imaging) studies of migraine attacks in the aura stage (Schulte and May, 2016; Schulte et al., 2020) discovered that the hypothalamus and brainstem structures (related to sleep and biology) were activated, thus possibly leading to sleep disturbance through a common neural pathway. In addition, we speculate that the occurrence of aura symptoms may add to the sufferers’ psychological pressure and thus indirectly affect their sleep quality. Anxiety is also a risk factor for poor sleep quality. Migraine sufferers with anxiety are 2.778 times more likely to have poor sleep quality than those without anxiety, which is consistent with the findings of Zhu et al. (2013). Previous studies have proved that prefrontal damage exists in most anxious patients, and adolescents with anxiety disorders exhibit decreased non-rapid eye movement sleep (NREM Sleep), long night sleep latency, more awakenings (Forbes et al., 2008), and EEG slow-wave activities (Sysoeva and Verbitsky, 2013). These changes in the sleep stages are also accompanied by decreases in subjective sleep quality (Papadimitriou and Linkowski, 2005) and objective sleep efficiency (Forbes et al., 2008), suggesting that poor sleep quality is positively correlated with high levels of anxiety (Ben Simon et al., 2020). The influences of anxiety on sleep quality have been confirmed by previous studies, and migraine itself may also affect sleep quality (Seidel et al., 2009), so it is quite possible that the sleep quality of students with migraine and anxiety disorders will decrease, which supports our findings. At the same time, sleep disturbances can also exacerbate most anxiety disorders which indicate a close relationship between the two.

We found that the MPAI score was correlated with sleep quality. The average MPAI score of the poor sleep quality group was higher than that of the good sleep quality group. The high MPAI score, equivalent to heavy dependence on mobile phones, indicates the high possibility of poor sleep quality. According to Demir and Sumer (2019), smartphone use increases headache duration and frequency of migraine sufferers, and excessive use is correlated with poor sleep quality and daytime sleepiness, ultimately leading to poor life quality. According to Demirci et al. (2015), excessive mobile phone use may lead to anxiety or depression, which in turn court sleep problems. This research is largely consistent with the findings in this study. Smartphones are extremely common among students. According to the survey results, for students with migraine, mobile phone use for recreation and interpersonal needs accounts for about 70%, and more than 70% of students with migraine spend an average of more than 4 h per day on mobile phones. Therefore, it is incumbent upon us to inform them of the negative impacts of excessive mobile phone use on sleep, insomuch that they can avoid excessive dependence on mobile phones and their sleep quality can be improved accordingly.

In this study, there was no difference in the sleep quality of male and female migraine sufferers (*p* > 0.05). The results of binary logistic regression analysis showed that aura symptoms, anxiety, and MPAI score were the factors that influenced the sleep quality of migraine sufferers, but we found discrepancies when we further analyzed the factors by sex. For the male migraine sufferers, the risk of poor sleep quality increased by 1.667 times, 1.957 times, and 1.015 times, respectively when they were diagnosed with aura symptoms, anxiety, and mobile phone addiction, but the data were not statistically significant (*p* > 0.05), which illustrates that for male migraine sufferers, these factors fail to affect their sleep quality. However, for female migraine sufferers, the risk of poor sleep quality increased by 3.796 times, 3.146 times, and 1.028 times, respectively when they were diagnosed with aura symptoms, anxiety, and mobile phone addiction. The risk results were of statistical significance (*p* > 0.05) and consistent with the overall regression analysis results of students with migraine. What is more, the OR for each factor in the female migraine population was higher than that in the overall migraine population. The discrepancies in contributing factors due to sex suggest that in clinical work, different emphasis needs to be put on the sleep quality of male and female migraine patients, so more targeted treatment plans can be provided.

Interestingly, the prevalence of depression in the poor sleep quality group was higher than that in the good sleep quality group. In the univariate logistic regression analysis, depression affected sleep quality, but the multivariate logistic regression analysis results showed that depression had no impact on sleep quality, which contrasts with the findings of outpatients with migraine in Southwest China (Zhu et al., 2013). Some scholars believed that the poor sleep quality of migraine patients is the result of the disease itself and cannot be fully explained by psychiatric comorbidities (Seidel et al., 2009). We believe, however, that depression may lead to poor sleep quality by adding to the migraine sufferers’ stress which was found to be a predictive factor of poor sleep quality in the general population (Hu et al., 2020). Furthermore, as the survey participants are medical students, they are more likely to seek help when they are liable to depression, thereby only 0.9% of the participants with severe depression in the study, which may explain why depression failed to influence the sleep quality in our study. Although we found statistically significant differences in headache frequency between sociodemographic data and clinical features, the results of multivariate logistic regression analysis revealed that headache frequency showed no effect on the sleep quality of students with migraine, which is inconsistent with the study results of migraine outpatients in Taiwan (Lin et al., 2016). The inconsistency is possibly caused by differences in age groups and research methods.

Our study has certain limitations. For example, we conducted a cross-sectional study on migraine-related characteristics with the survey form questionnaire, and there may exist reporting biases and unavoidable recall biases, so we cannot further explore whether there is a causal relationship between poor sleep quality and its risk factors. We did not analyze the sleep quality of the non-migraine college student population, meaning that we could not compare the prevalence of poor sleep quality among college students with/without migraine and to explore how migraine contributes to poor sleep quality. Besides, our study only found that aura symptoms, anxiety, and MPAI score were the factors that influence the sleep quality of migraine sufferers (especially female sufferers), but we failed to discover the factors that influence the sleep quality of male migraine sufferers, which deserves further exploration. The examination schedules of each semester may affect the quality of students’ sleep, but this factor was not addressed in this study. We did not further describe the type of aura and the proportion of chronic migraine. Moreover, the study failed to further analyze how significant the effect of each factor is, which is also one of the limitations.

## Conclusions

In conclusion, our study discovers that the prevalence of migraine among students at North Sichuan Medical College is 6.6%, and the incidence of poor sleep quality among students with migraine is up to 64.04%. Such factors as anxiety, aura symptoms, and MPAI score are the risk factors affecting sleep quality, while depression, headache frequency, headache intensity, and duration show no influence on sleep quality. More than 70% of the participants with migraine spend an average of more than 4 h per day on mobile phones, and the avoidance of mobile phone addiction will help improve sleep quality and relieve headaches. Poor sleep quality seriously affects the study and life of students with migraine, so appropriate prevention and intervention measures are of great significance to improve their physical and mental health.

## Data Availability Statement

The raw data supporting the conclusions of this article will be made available by the authors, without undue reservation.

## Ethics Statement

The studies involving human participants were reviewed and approved by the Ethics Committee of Affiliated Hospital of North Sichuan Medical College, and the project number was 2017ER (R) 040. The patients/participants provided their written informed consent to participate in this study.

## Author Contributions

JZ, YC, and JiaL contributed to design the research study, manuscript conceptualization, methodology, writing original draft and translation. JiaL and JW contributed to funding acquisition, project administration and supervision. JY, CL, YL, ZR, and YX contributed to investigation and data collection and curation. JH, JinL, YZ, and WL analyzed the data and provided critical revisions. All authors are accountable for all aspects presented in this study. All authors contributed to the article and approved the submitted version.

## Funding

This work was supported by the Funding Project of the Bureau of Science and Technology and Intellectual Property of Nanchong City (No. NSMC20170420 and No. 19SXHZ0023).
